# The Combination of Feature Tracking and Late Gadolinium Enhancement for Identification Between Hypertrophic Cardiomyopathy and Hypertensive Heart Disease

**DOI:** 10.3389/fcvm.2022.865615

**Published:** 2022-05-11

**Authors:** Shengliang Liu, Yunling Li, Yanming Zhao, Xueying Wang, Zhiyuan Wu, Xia Gu, Bing Xu, Ye Li, Jinwei Tian, Jinjin Cui, Guokun Wang, Bo Yu

**Affiliations:** ^1^Department of Cardiology, Cardiovascular Imaging Center, The Second Affiliated Hospital of Harbin Medical University, Harbin, China; ^2^Department of Vascular Surgery, Beijing Hospital, National Center of Gerontology, Beijing, China; ^3^Institute of Geriatric Medicine, Chinese Academy of Medical Sciences, Beijing, China; ^4^Department of Cardiology, The Second Affiliated Hospital of Harbin Medical University, Harbin, China; ^5^The Key Laboratory of Myocardial Ischemia, Chinese Ministry of Education, Harbin, China

**Keywords:** hypertrophic cardiomyopathy, hypertensive heart disease, cardiovascular resonance magnetic, feature tracking, late gadolinium enhancement

## Abstract

**Background:**

The differentiation between hypertrophic cardiomyopathy (HCM) and hypertensive heart disease (HHD) is challenging due to similar myocardial hypertrophic phenotype. The purpose of this study is to evaluate the feasibility of cardiovascular magnetic resonance feature tracking (CMR-FT) and late gadolinium enhancement (LGE) to distinguish between HCM and HHD and the potential relationship between myocardial strain and cardiac functional parameters.

**Methods:**

One hundred and seventy subjects (57 HCM, 45 HHD, and 68 controls) underwent 3.0 T CMR, including steady-state free precession cines and LGE images. Global and segmental (basal, mid, and apical) analyses of myocardial radial, circumferential, longitudinal strain, and left ventricular (LV) torsion, as well as global and 16 segments of LGE were assessed. The multivariate analysis was used to predict the diagnostic ability by combining comprehensive myocardial strain parameters and LGE.

**Results:**

Global radial strain (GRS), global circumferential strain (GCS), and LV torsion were significantly higher in the HCM group than in the HHD group (GRS, 21.18 ± 7.52 vs. 14.56 ± 7.46%; GCS, −13.34 ± 3.52 vs. −10.11 ± 4.13%; torsion, 1.79 ± 0.69 vs. 1.23 ± 0.65 deg/cm, all *P* < 0.001). A similar trend was also seen in the corresponding strain rate. As for segmental strain analysis, basal radial strain (BRS), basal circumferential strain (BCS), basal longitudinal strain (BLS), mid-radial strain (MRS), and mid-circumferential strain (MCS) were higher in the HCM group than in the HHD group (all *P* < 0.001). The receiver operating characteristic (ROC) results showed that the area under the curve (AUC) of LGE in the mid-interventricular septum (mIVS) was the highest among global and segmental LGE analyses. On the multivariate regression analysis, a combined model of LGE (mIVS) with GRS obtained the highest AUC value, which was 0.835 with 88.89% sensitivity and 70.18% specificity, respectively. In addition, for patients with HCM, GRS, GCS, and global longitudinal strain had correlations with LV ejection fraction (LVEF), maximum interventricular septum thickness (IVST max), and left ventricular mass index (LVMi). Torsion was mildly associated with LVEF.

**Conclusion:**

CMR-FT-derived myocardial strain and torsion provided valuable methods for evaluation of HCM and HHD. In addition, the combination of GRS and LGE (mIVS) achieved the highest diagnostic value.

## Introduction

Hypertrophic cardiomyopathy (HCM) is the most common genetically transmitted cardiomyopathy (1). HCM is diagnosed according to the European Society of Cardiology (ESC) guidelines with unexplained left ventricular (LV) hypertrophy (LVH) and a maximal wall thickness ≥ 15 mm (2). Hypertensive heart disease (HHD) is characterized by extensive structural remodeling with a dilated LV cavity and increased LV wall thickness (LVWT) as a result of enhanced arterial pressure (3). Both the HCM and HHD are associated with LVH (2, 4), which can make their differentiation challenging. The increase in LVWT can be formed by increased afterload, changes of the myocardial structure, and alterations caused by genetic defects or infiltrative disease (5). Treatment and risk stratification of the two diseases are totally different; therefore, it is particularly vital and necessary to diagnose and distinguish between these two diseases.

Cardiovascular magnetic resonance (CMR) is the gold standard for evaluation of cardiac morphology, function, and tissue characterization. The presence of late gadolinium enhancement (LGE) represents changes in fibrosis or myocardial scarring, which can significantly predict the prognosis of severe cardiac complications (6). A previous study detected more prevalent mid-wall LGE in patients with HCM relative to patients with HHD (7). Another study demonstrated that LGE served as a significant diagnostic index to discriminate between HCM and HHD by using a semiquantitative score system (5). The interventricular septum (IVS) is the most predominantly hypertrophied segment in HCM disease. While the IVS is also involved in HHD, LV wall thickness tends to be concentric rather than asymmetric for this cohort. Evaluation of LGE in the mid-wall of the IVS might offer novel insights to better characterize these two hypertrophied cohorts.

Cardiovascular magnetic resonance feature tracking (CMR-FT) technology allows for high-resolution evaluation of myocardial deformation and the determination of myocardial strain parameters with excellent consistency and reproducibility (8–10). It tracks tissue motion between epicardial and endocardial borders and measures changes of cardiac dimensions throughout the whole cardiac cycle, thus potentially distinguishing the normal and abnormal cardiac functions (11). Global longitudinal strain (GLS) was significantly higher in the HCM group than in the HHD group and the circumferential strain difference between the endocardium and epicardium has been shown to identify preclinical and overt HCM (8, 12). However, most studies depicted cardiac systolic dysfunction at a global level, rather than at a regional myocardial function level.

Left ventricular torsion obtained from CMR-FT can be used to evaluate the ability of rotation during left ventricular motion, which plays a crucial role in pumping blood out of the heart. As for patients with dilated cardiomyopathy, amplitude of torsion was lower than that in healthy subjects and it was associated with LVEF and clinical endpoints (13, 14). However, LV torsion has not been widely used to characterize HCM and HHD. Moreover, limited studies focused on comprehensive strain analysis for differentiating HCM from HHD, especially the lack of combination with LGE images.

The purpose of this study is to evaluate the feasibility of CMR-FT and LGE to distinguish between HCM and HHD and to investigate the potential relationship between CMR-FT strain and cardiac functional parameters.

## Methods

A systematic retrospective analysis was conducted in one hundred and seventy consecutive subjects who underwent CMR from September 2020 to November 2021. All the images exhibited diagnostic quality, enabling the assessment of myocardial strain and LGE. Three cohorts were recruited: (i) 68 healthy volunteers who were free of any history of medical conditions; (ii) 57 patients with HCM, which was defined as nondilated left ventricular hypertrophy with end-diastolic wall thickness ≥ 15 mm (or ≥ 13 mm whose first-degree relatives had diagnosis of HCM) according to current established CMR diagnostic criteria (2); and (iii) 45 patients with HHD, which was defined as increased LVWT (≥12 mm) in the context of concomitant systemic hypertension (3). The exclusion criteria included: subjects with other diseases that accounted for increased LVWT, such as aortic valve disease, infiltrative disease (cardiac amyloidosis, Anderson–Fabry disease, and Danon disease), and systemic disease. Patients with MRI conventional contraindications were also excluded, such as implanted pacemakers, metallic intracranial implants, claustrophobia, and renal function impairment. Apical HCM was excluded, as it was readily differentiated from HHD. This study was approved by local ethics committee and all the subjects gave a written informed consent.

Cardiovascular MRI was performed with a 3.0-Tesla system (Ingenia CX, Philips Healthcare, The Netherlands) using a 32-channel phased-array abdomen coil. Cine images were performed by using a steady-state free precession (SSFP) sequence with a breath-hold and ECG trigger for cardiac morphologic and functional analyses. The scanning parameters were as follows: repetition time (TR)/echo time (TE) = 2.8/1.42 ms, field of view (FOV) = 300 × 300 mm^2^, voxel = 1.8 × 1.6 × 8.0 mm, flip angle = 45°, and 8-mm slice thickness. LGE images were acquired 10 min after intravenous injection of 0.1 mmol/kg of gadolinium-based contrast agent (Bayer Healthcare, Germany) by using a three-dimensional phase-sensitive inversion recovery (PSIR) sequence; the scanning parameters were as follows: TR/TE = 6.1/3.0 ms, FOV = 300 × 300 mm^2^, voxel = 1.8 × 1.68 × 8.0 mm, flip angle = 25°, and 8-mm slice thickness. The acquisitions of SSFP cines and PSIR were conducted in 2-chamber, 3-chamber, and 4-chamber long-axis planes, as well as a stack of contiguous short-axis slices, which encompassed the left ventricular from the atrioventricular ring to the apex.

All the CMR studies were postprocessed using a commercially available workstation (cvi42, Circle Cardiovascular Imaging Incorporation, Calgary, Alberta, Canada). LV endocardial and epicardial borders were delineated automatically with manual calibrations throughout the cardiac cycle by a radiologist (with 3 years of experience in MRI) who was blinded to clinical information. LVEF, cardiac output (CO), end-diastolic volume (EDV), end-systolic volume (ESV), interventricular septal thickness (IVST), and cardiac chamber dimensions were measured. LV mass was calculated as the total myocardium volume multiplied by myocardial gravity (1.05 g/ml) without including papillary muscles.

**Figure 1:**
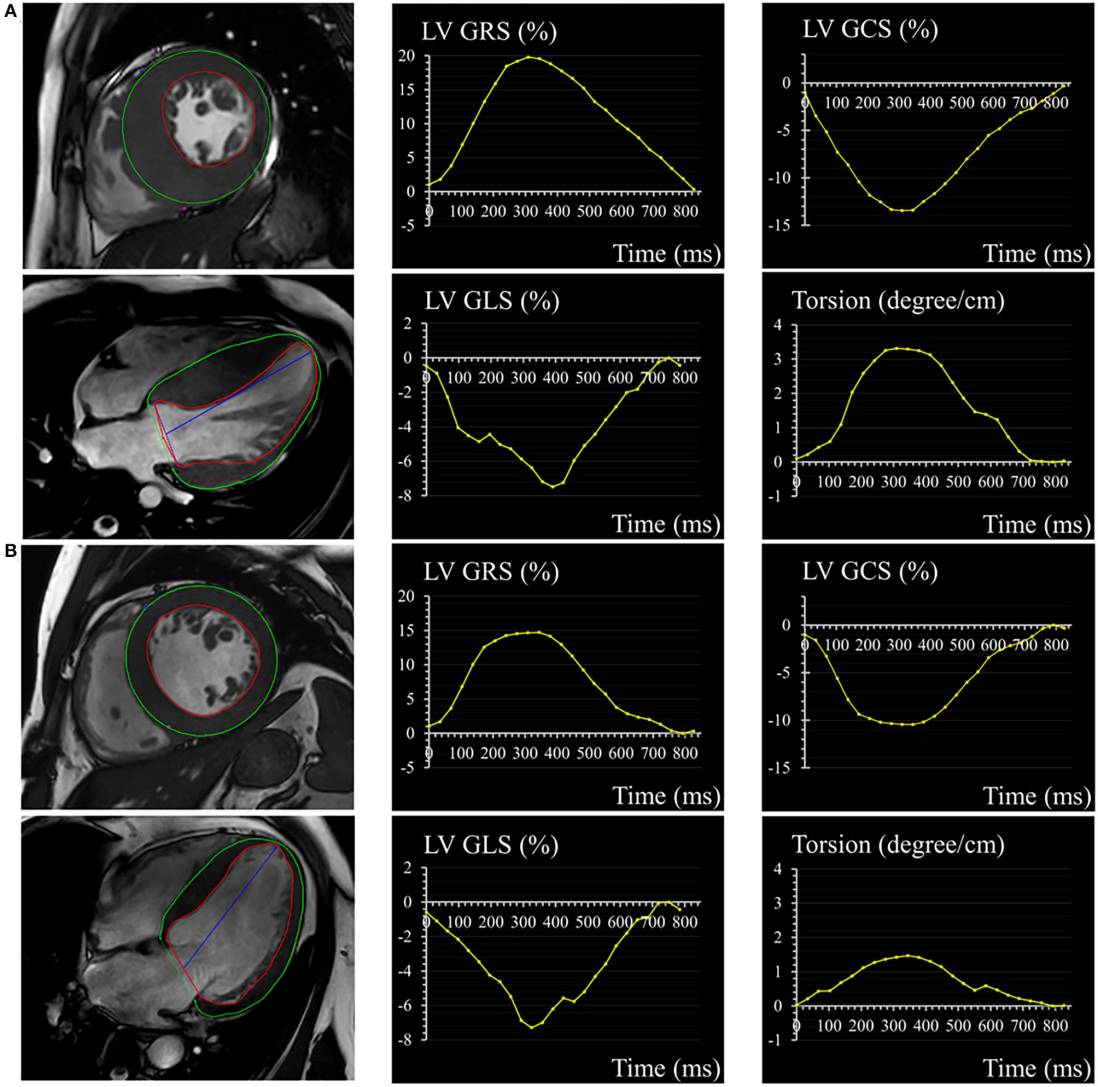
A 32-year-old male with HCM **(A)** and a 56-year-old male with HHD **(B)**. Epicardial (green) and endocardial (red) contours, as well as the corresponding curves of LV GRS, GCS, GLS, and torsion in HCM and HHD. HCM, hypertrophic cardiomyopathy; HHD, hypertensive heart disease; LV, left ventricular; GRS, global radial strain; GCS, global circumferential strain; GLS, global longitudinal strain.

Cardiovascular magnetic resonance feature tracking-derived strain and strain rate were used to evaluate myocardial deformation. Quantitative parameters included radial, circumferential, and longitudinal orientation (Figure 1). Radial strain and circumferential strain were calculated from two-dimensional short-axis planes and longitudinal strain was derived from two-dimensional long-axis plane. Regional strain analysis was also performed by dividing left ventricular into basal, mid, and apical segments by 2-dimension. Strain rate was measured as a derivative of the strain tensor and it represented the rate of deformation (15). The LV torsion was calculated as the ratio of the peak difference of ventricular apical and basal rotation at the same time point in the cardiac cycle to the distance between their short-axis slices (13).

Late gadolinium enhancement quantification was obtained with mean ± 5 SDs algorithm and expressed as a volumetric proportion of the total LV myocardium. Segmental analysis on LGE was assessed according to the American Heart Association 17-segment model by setting the anterior and inferior right ventricular insertion points (16). To investigate the difference of LGE in different segments of myocardium in HCM and HHD, the segments 8 and 9 were defined as mid-interventricular septal (mIVS) in this study.

Categorical variables were assessed by the Fisher's exact test or the chi-squared test and expressed as numbers with percentage. Normally, distributed continuous variables were presented as mean ± SD and compared by the Student's *t*-test between the two groups or by ANOVA test with *post-hoc* Bonferroni analysis among the three groups. Nonnormally distributed continuous variables were shown as medians with interquartile range and verified by the Mann–Whitney *U* test or the Kruskal–Wallis *H* test. The Pearson's or Spearman's correlation coefficients were calculated to investigate the potential correlations between myocardial strain and functional CMR parameters. The multivariate logistic regression analysis was calculated to test the diagnostic ability of CMR parameters for distinguishing the two diseases. The area under the curves (AUCs), specificity, sensitivity, and Youden's index were analyzed by the receiver operating characteristic (ROC) curve. DeLong's test was used to compare the AUCs for the pairwise groups. All the data were calculated by SPSS version 26.0.0 (SPSS Incorporation, Chicago, Illinois, USA) or MedCalc (version 20, MedCalc Software, Ostend, Belgium). *P* < 0.05 was considered as statistically significant.

## Results

### Participant Characteristics

The study cohort consisted of 170 participants, including 57 patients with HCM (33.5%), 45 patients with HHD (26.5%), and 68 healthy volunteers (40%). Clinical characteristics and basic CMR parameters of the HCM, HHD, and healthy group are given in Table 1. In comparison to patients with HHD, patients with HCM were older (*P* = 0.014), less male (*P* = 0.009), had smaller body surface area (*P* < 0.001), and lower incidence of hypertension (*P* < 0.001). There was no statistical difference between the HCM and HHD group in terms of prevalence of diabetes mellitus, dyslipidemia, and smoker (all *P* > 0.05).

**Table 1:** Demographic data and baseline characteristics of the study population.

	**HCM (*n* = 57)**	**HHD (*n* = 45)**	^*^ *P*	**Healthy (*n* = 68)**	**^§^ *P***
Age (years)^#^	55.33 ± 10.11	47.78 ± 15.03	0.014	52.38 ± 15.29	0.023
BSA (m^2^)^#^	1.75 ± 0.21	1.94 ± 0.23	<0.001	1.74 ± 0.20	<0.001
Sex, male *n* (%)^‡^	33 (57.9)	37 (82.2)	0.009	32 (47.1)	0.001
Hypertension, *n* (%)^‡^	29 (50.9)	45 (100)	<0.001	15 (22.1)	<0.001
DM, n (%)^‡^	7 (12.3)	8 (17.8)	0.436	7 (10.3)	0.502
Dyslipidemia, *n* (%)^‡^	24 (42.1)	24 (53.3)	0.259	27 (39.7)	0.336
Smoker, *n* (%)^‡^	21 (36.8)	21 (46.7)	0.317	15 (22.1)	0.020
LV EF (%)^#^	64.43 ± 13.82	39.76 ± 18.59	<0.001	66.45 ± 7.01	<0.001
LV CO (L/min)^#^	6.47 ± 2.06	6.20 ± 1.74	0.718	5.81 ± 1.50	0.113
LV EDV (ml)^#^	148.19 ± 54.97	240.18 ± 103.23	<0.001	128.83 ± 27.59	<0.001
LV EDVi (ml/m^2^)^#^	84.83 ± 35.16	121.08 ± 46.36	<0.001	73.45 ± 14.89	<0.001
LV ESV (ml)^pounds^	46.3 (34.2, 60.8)	154.6 (56.4, 213.3)	<0.001	44.4 (33.6, 52.6)	<0.001
LV ESVi (ml/m^2^)^pounds^	26.3 (19.3, 32.8)	81.6 (30.6, 107.7)	<0.001	24.9 (19.8, 29.9)	<0.001
LV mass (g)^#^	151.63 ± 50.18	182.71 ± 49.71	0.007	82.96 ± 21.44	<0.001
LVMi (g/m^2^)^#^	86.38 ± 25.31	93.77 ± 20.67	0.241	47.25 ± 9.14	<0.001
IVST min (mm)^#^	11.05 ± 4.56	10.68 ± 2.18	0.850	6.42 ± 1.56	<0.001
IVST max (mm)^#^	23.40 ± 4.48	15.86 ± 3.73	<0.001	10.04 ± 2.36	<0.001
LV EDD (mm)^#^	45.75 ± 7.39	60.73 ± 11.59	<0.001	47.16 ± 4.33	<0.001
RV EDD (mm)^#^	27.91 ± 6.99	29.96 ± 8.50	0.361	27.82 ± 7.21	0.278

* *P is for HCM vs. HHD*.

§ *P is for HCM and HHD patients vs. healthy volunteers*.

# *Data are means ± standard deviation*.

‡ *Data are numbers, with percentages in parentheses*.

*^pounds^Data are medians with interquartile. P < 0.05 is considered to indicate statistical significance. DM, Diabetes mellitus; BSA, body surface area; LV, left ventricular; EF, ejection fraction; CO, cardiac output; EDV, end diastolic volume; EDVi, end diastolic volume index; ESV, end systolic volume; ESVi, end systolic volume index; LVMi, left ventricular mass index; IVST, interventricular septum thickness; EDD, end diastolic diameter; RV, right ventricular*.

### Cardiac Function by Cardiovascular Magnetic Resonance

Compared with patients with HHD, patients with HCM had a higher ejection fraction (64.43 ± 13.82 vs. 39.76 ± 18.59%, *P* < 0.001). Those with HCM also had a lower LV end-diastolic volume index (EDVi) (84.83 ± 35.16 vs. 121.08 ± 46.36 ml/m^2^, *P* < 0.001) and lower LV end-systolic volume index (ESVi) [26.3 (19.3, 32.8) vs. 81.6 (30.6, 107.7) ml/m^2^, *P* < 0.001]. As for maximum interventricular septum thickness (IVST max), there were higher values in patients with HCM when compared with patients with HHD (23.40 ± 4.48 vs. 15.86 ± 3.73 mm, *P* < 0.001). The LV end-diastolic diameter (EDD) of patients with HCM was 45.75 ± 7.39 mm, which was lower than that in patients with HHD (60.73 ± 11.59 mm, *P* < 0.001). There were no significant differences regarding CO, LVMi, and right ventricular EDD between the two groups of LVH.

### Myocardial Strain and Late Gadolinium Enhancement in the Hypertrophic Cardiomyopathy and Hypertensive Heart Disease Groups

Global radial strain (GRS) and global circumferential strain (GCS) were significantly higher in the HCM group relative to the HHD group, as shown in Table 2 (GRS, 21.18 ± 7.52 vs. 14.56 ± 7.46%; GCS, −13.34 ± 3.52 vs. −10.11 ± 4.13%; both *P* < 0.001), whereas comparisons of GLS showed no obvious difference (−9.49 ± 3.06 vs. −9.34 ± 3.76%, *P* = 0.973). Similar trend could also be detected in corresponding strain rate.

**Table 2:** CMR strain and LGE data of the study population.

	**HCM (*n* = 57)**	**HHD (*n* = 45)**	^*^ *P*	**Healthy (*n* = 68)**	**^§^ *P***
GRS (%)^#^	21.18 ± 7.52	14.56 ± 7.46	<0.001	32.43 ± 6.09	<0.001
GCS (%)^#^	−13.34 ± 3.52	−10.11 ± 4.13	<0.001	−18.66 ± 2.18	<0.001
GLS (%)^#^	−9.49 ± 3.06	−9.34 ± 3.76	0.973	−17.60 ± 1.57	<0.001
GRS rate (1/s)^#^	1.27 ± 0.53	0.89 ± 0.44	<0.001	1.64 ± 0.42	<0.001
GCS rate (1/s)^#^	−0.83 ± 0.28	−0.64 ± 0.25	0.008	−0.95 ± 0.38	<0.001
GLS rate (1/s)^#^	−0.61 ± 0.21	−0.55 ± 0.26	0.428	−0.91 ± 0.15	<0.001
BRS (%)^#^	24.12 ± 8.21	15.25 ± 7.88	<0.001	34.45 ± 6.95	<0.001
BCS (%)^#^	−14.73 ± 3.61	−10.41 ± 4.31	<0.001	−19.26 ± 2.38	<0.001
BLS (%)^#^	−15.93 ± 4.51	−13.55 ± 5.02	0.039	−22.34 ± 2.87	<0.001
MRS (%)^#^	19.90 ± 7.83	13.77 ± 8.27	<0.001	31.12 ± 6.73	<0.001
MCS (%)^#^	−12.89 ± 3.76	−9.70 ± 4.68	0.001	−18.28 ± 2.52	<0.001
MLS (%)^#^	−6.69 ± 4.38	−7.89 ± 3.97	0.323	−15.19 ± 2.86	<0.001
ARS (%)^#^	22.70 ± 12.62	18.24 ± 10.14	0.124	35.25 ± 11.04	<0.001
ACS (%)^#^	−13.26 ± 5.37	−11.73 ± 4.79	0.216	−19.10 ± 3.58	<0.001
ALS (%)^#^	−7.96 ± 5.52	−8.14 ± 4.57	0.982	−14.98 ± 3.31	<0.001
Torsion (deg/cm)^#^	1.79 ± 0.69	1.23 ± 0.65	<0.001	1.45 ± 0.71	<0.001
LGE (%)^pounds^	7.4 (2.4, 13.9)	4.0 (1.9, 7.5)	0.040	0	<0.001
LGE Segment 7 (%)^pounds^	4.4 (1.0, 14.5)	1.0 (0.2, 4.1)	0.001	0	<0.001
LGE Segment 8 (%)^pounds^	4.6 (0.8, 14.7)	0.7 (0.2, 3.5)	0.001	0	<0.001
LGE Segment 9 (%)^pounds^	4.5 (1.3, 11.3)	1.2 (0.5, 3.7)	0.001	0	<0.001
LGE mIVS (%)^pounds^	5.4 (1.5, 15.9)	1.3 (0.4, 3.4)	<0.001	0	<0.001

* *P is for HCM vs. HHD*.

§ *P is for HCM and HHD patients vs. healthy volunteers*.

# *Data are means ± standard deviation*.

*^**pounds**^Data are medians with interquartile range. P < 0.05 is considered to indicate statistical significance. CMR, cardiovascular magnetic resonance; GRS, global radial strain; GCS, global circumferential strain; GLS, global longitudinal strain; BRS, basal radial strain; BCS, basal circumferential strain; BLS, basal longitudinal strain; MRS, mid radial strain; MCS, mid circumferential strain; MLS, mid longitudinal strain; ARS, apical radial strain; ACS, apical circumferential strain; ALS, apical longitudinal strain; LGE, late gadolinium enhancement; mIVS, mid interventricular septum*.

As for segmental strain analysis, the three basal parameters [which included basal radial strain (BRS), basal circumferential strain (BCS), and basal longitudinal strain (BLS)] were higher in the HCM group than in the HHD group (BRS, 24.12 ± 8.21 vs. 15.25 ± 7.88%; BCS, −14.73 ± 3.61 vs. −10.41 ± 4.31%; BLS, −15.93 ± 4.51 vs. −13.55 ± 5.02%; all *P* < 0.05). Mid-radial strain (MRS) and mid-circumferential strain (MCS) were also higher in patients with HCM relative to patients with HHD (MRS, 19.9 ± 7.83 vs. 13.77 ± 8.27%; MCS, −12.89 ± 3.76 vs. −9.7 ± 4.68%; both *P* < 0.05), but mid-longitudinal strain (MLS) had no significant difference between the two groups. None of the three apical strain results showed obvious differences between the HCM and HHD cohort. Torsion in the HCM group was higher than that in the HHD group (1.79 ± 0.69 vs. 1.23 ± 0.65 deg/cm, *P* < 0.001).

**Figure 2:**
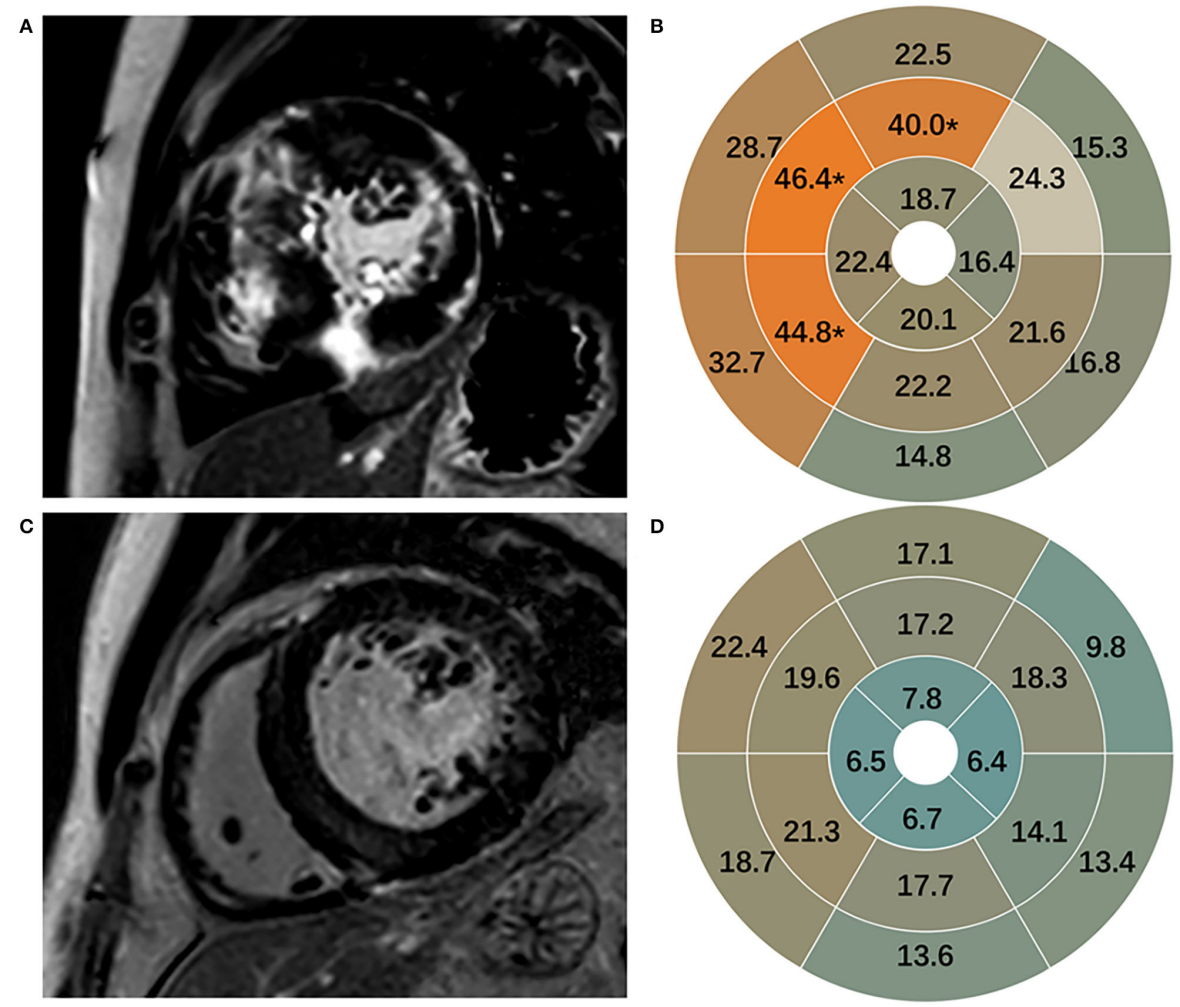
Representative images of LGE in HCM **(A)** and HHD **(C)**. LGE in 16 segments in HCM **(B)** and HHD **(D)**. LGE, late gadolinium enhancement; HCM, hypertrophic cardiomyopathy; HHD, hypertensive heart disease.

Late gadolinium enhancement was assessed in 170 participants and shown in Table 2. Healthy subjects presented no LGE. Patients with HCM in comparison to patients with HHD had higher values of LGE (total enhanced volume percentage) (*P* < 0.05). On segmental analysis, the differences in LGE values were predominantly localized around the interventricular septum (segments 7, 8, and 9; all *P* = 0.001), which corresponds to the mid-segment of the anterior, anteroseptal, and inferoseptal of IVS. It was also pronounced for mIVS (*P* < 0.001) (Figure 2), but there were no significant differences in other segments.

### Correlations of Left Ventricular Strain With Functional and Structural Parameters

**Figure 3:**
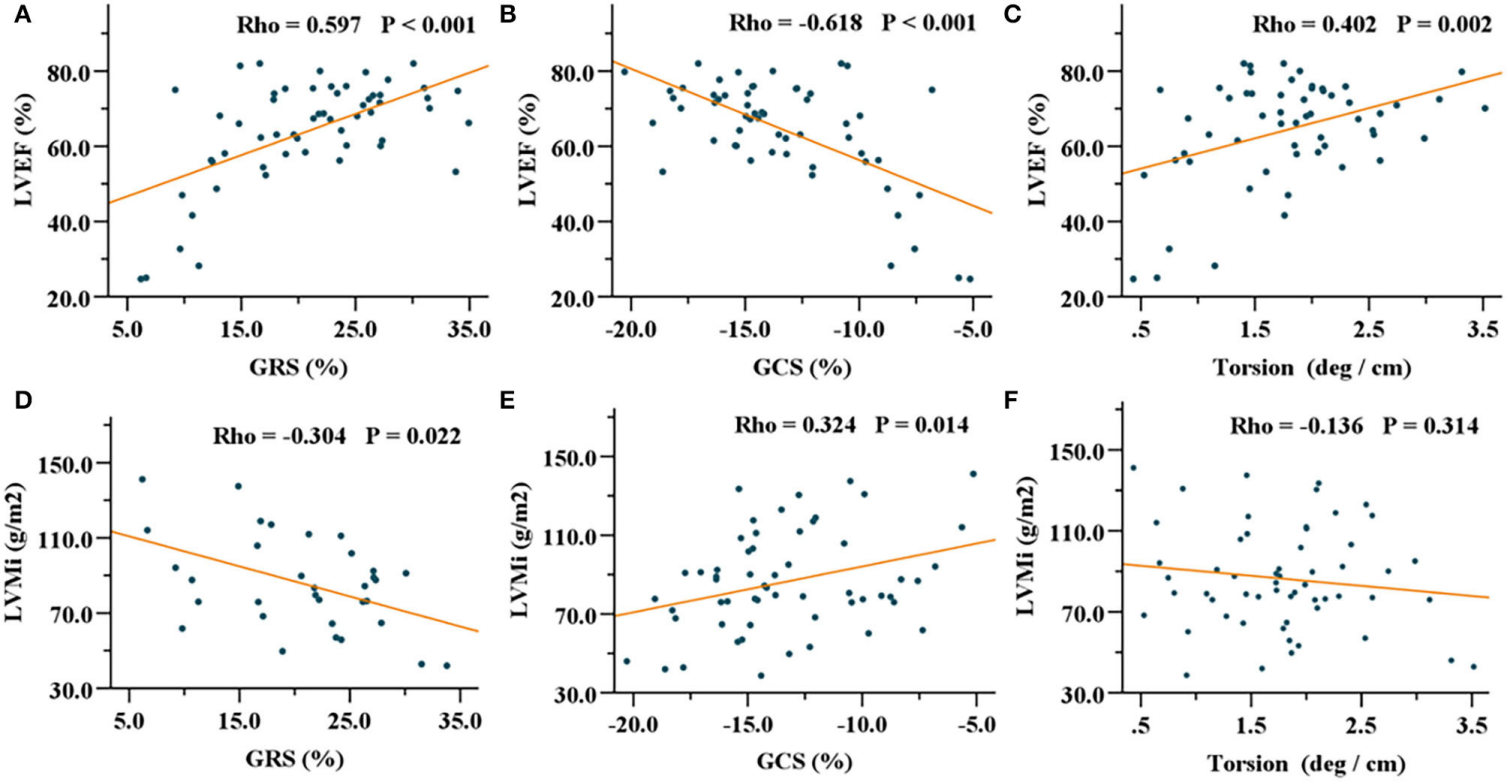
Correlations of GRS, GCS, and torsion with LVEF **(A–C)** and LVMi **(D–F)** in the HCM group. GRS, global radial strain; GCS, global circumferential strain; LVEF, left ventricular ejection fraction; LVMi, left ventricular mass index; HCM, hypertrophic cardiomyopathy.

In patients with HCM, GRS, GCS, and GLS showed correlations with LVEF, IVST max, and LVMi (Table 3). Especially, GRS and GCS presented strong correlations with LVEF (GRS, *R* = 0.597, *P* < 0.001; GCS, *R* = −0.618, *P* < 0.001). In addition, torsion was mildly associated with LVEF (*R* = 0.402, *P* = 0.002) (Figure 3). In patients with HHD, GRS, GCS, GLS, and torsion were correlated with LVEF and LVMi (all *P* < 0.001). In the healthy group, GRS, GCS, and torsion were only associated with LVEF (all *P* < 0.001).

**Table 3:** Correlations of left ventricular strain with functional and structural parameters.

	**LVEF (%)**		**IVST max (mm)**		**LVMi (g/m** ^**2**^ **)**	
	**Rho**	***P***	**Rho**	***P***	**Rho**	***P***
HCM						
GRS (%)	0.597	<0.001	−0.297	0.025	−0.304	0.022
GCS (%)	−0.618	<0.001	0.308	0.020	0.324	0.014
GLS (%)	−0.343	0.009	0.486	<0.001	0.432	0.001
Torsion (deg/cm)	0.402	0.002	−0.211	0.115	−0.136	0.314
HHD						
GRS (%)	0.929	<0.001	0.107	0.486	−0.639	<0.001
GCS (%)	−0.937	<0.001	−0.093	0.545	0.662	<0.001
GLS (%)	−0.840	<0.001	0.084	0.584	0.675	<0.001
Torsion (deg/cm)	0.769	<0.001	0.067	0.663	−0.547	<0.001
Healthy						
GRS (%)	0.578	<0.001	0.151	0.220	0.035	0.777
GCS (%)	−0.549	<0.001	−0.118	0.338	−0.011	0.928
GLS (%)	−0.095	0.443	0.187	0.128	0.182	0.138
Torsion (deg/cm)	0.429	<0.001	0.392	0.001	0.115	0.348

*P < 0.05 is considered to indicate statistical significance. HCM, hypertrophic cardiomyopathy; HHD, hypertensive heart disease; GRS, global radial strain; GCS, global circumferential strain; GLS, global longitudinal strain; LVEF, left ventricular ejection fraction; IVST, interventricular septum thickness; LVMi, left ventricular mass index*.

### Differentiation Between Hypertrophic Cardiomyopathy and Hypertensive Heart Disease

**Figure 4:**
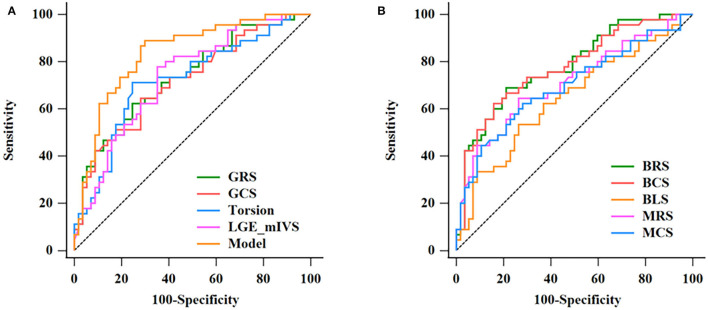
Discrimination of HCM and HHD by CMR parameters. The ROC curves in discrimination between HCM and HHD for single CMR parameters and the multivariate regression model are given in Table 5 **(A)**. The AUC of combination model was the highest (0.835). The ROC curves of BRS, BCS, BLS, MRS, MCS in discrimination between HCM and HHD group **(B)**. HCM, hypertrophic cardiomyopathy; HHD, hypertensive heart disease; ROC, receiver operating characteristic; CMR, cardiovascular magnetic resonance; AUC, area under the curve; GRS, global radial strain; GCS, global circumferential strain; LGE, late gadolinium enhancement; mIVS, mid interventricular septum; BRS, basal radial strain; BCS, basal circumferential strain; BLS, basal longitudinal strain; MRS, mid radial strain; MCS, mid circumferential strain.

The results of the ROC analysis to discriminate between HCM and HHD are shown in Table 4. GRS (AUC: 0.734, sensitivity: 62.22%, specificity: 75.44%, *P* < 0.001), GCS (AUC: 0.718, sensitivity: 64.44%, specificity: 71.93%, *P* < 0.001), and corresponding strain rate could distinguish HCM from HHD. For segmental strain parameters, the AUCs of BRS, BCS, BLS, MRS, and MCS for discriminating between HCM and HHD were 0.781, 0.776, 0.644, 0.711, and 0.697, respectively (all *P* < 0.001). The torsion cutoff value of > 1.40 differentiated HCM from HHD with a sensitivity of 71.11% and a specificity of 75.44%. The AUC of LGE (mIVS) was 0.735, which was the highest in the global and other segmental LGE analyses (all *P* < 0.05). Overall, BRS showed the highest diagnostic performance and the value of the AUC was 0.781 (all *P* < 0.05). On the multivariate regression analysis, the combination model of LGE (mIVS) with GRS obtained the highest AUC value, which was 0.835 with 88.89% sensitivity and 70.18% specificity, respectively (Table 5). DeLong's test showed that the AUC of a combined model of GRS with LGE (mIVS) was higher than those of GRS, GCS, torsion, and LGE (mIVS) (all *P* < 0.05) (Figure 4).

**Table 4:** Results of the ROC for discrimination of patients with HCM and HHD.

**Parameters**	**AUC**	**Cut-off**	**Sensitivity (%)**	**Specificity (%)**	***P***
GRS (%)	0.734	16.27	62.22	75.44	<0.001
GCS (%)	0.718	−12.04	64.44	71.93	<0.001
GRS rate (1/s)	0.713	0.89	60.00	78.95	<0.001
GCS rate (1/s)	0.698	−0.62	51.11	84.21	<0.001
BRS (%)	0.781	18.09	68.89	78.95	<0.001
BCS (%)	0.776	−11.59	62.22	84.21	<0.001
BLS (%)	0.644	−13.45	53.33	73.68	0.010
MRS (%)	0.711	15.15	64.44	73.68	<0.001
MCS (%)	0.697	−11.14	62.22	71.93	<0.001
Torsion (deg/cm)	0.721	1.40	71.11	75.44	<0.001
LGE (%)	0.619	5.17	64.44	63.16	0.034
LGE Segment 7 (%)	0.684	2.10	66.67	66.67	<0.001
LGE Segment 8 (%)	0.701	3.93	80.00	54.39	<0.001
LGE Segment 9 (%)	0.699	4.20	82.22	54.39	<0.001
LGE mIVS (%)	0.735	3.43	77.78	64.91	<0.001

*P < 0.05 is considered to indicate statistical significance. ROC, receiver operating characteristic; HCM, hypertrophic cardiomyopathy; HHD, hypertensive heart disease; AUC, area under the curve; GRS, global radial strain; GCS, global circumferential strain; BRS, basal radial strain; BCS, basal circumferential strain; BLS, basal longitudinal strain; MRS, mid radial strain; MCS, mid circumferential strain; LGE, late gadolinium enhancement; mIVS, mid interventricular septum*.

**Table 5:** The multivariate logistic regression analysis of CMR parameters for discrimination of patients with HCM and HHD.

**Parameters**			**AUC**	**Sensitivity (%)**	**Specificity (%)**	***P***
Multivariate Analysis	Wald	OR (95% CI)				
LGE (mIVS) (%)	8.868	0.872^*^ (0.797, 0.954)	0.835	88.89	70.18	<0.001
GRS (%)	3.872	0.655^*^ (0.430, 0.998)				

*CMR, cardiovascular magnetic resonance; HCM, hypertrophic cardiomyopathy; HHD, hypertensive heart disease; AUC, area under the curve; OR: odds ratio; CI, confidence interval; LGE, late gadolinium enhancement; mIVS, mid interventricular septum; GRS, global radial strain*.

* *P < 0.05 on the multivariate regression analysis*.

## Discussion

Several important outcomes were found in evaluating patients with HCM and HHD using FT parameters and LGE data. The main findings of this study were: (1) LV GRS, GCS, and torsion were significantly higher in patients with HCM compared with patients with HHD. The differences of radial and circumferential strain mainly existed in LV basal and midsegments; (2) There was no significant difference in GLS between the two groups, but it was distinguishable in the LV basal segment (BLS); (3) The difference of %LGE can be used to distinguish between the two diseases, especially in the mIVS; and (4) In the multivariate regression model, the combination of GRS and LGE (mIVS) achieved the highest diagnostic value in differentiation between HCM and HHD diseases.

Cardiovascular magnetic resonance feature tracking has been used in the assessment of HCM and HHD, respectively, due to its ability to quantify cardiac movement in different directions during the cardiac cycle (17, 18). This study found that radial strain in the HHD group was decreased when compared with the HCM group and alterations of the two diseases mainly concentrated in the basal and midsegments. There were no obvious differences in the apical segment. Moreover, segmental analysis of radial strain offered excellent precision for the differentiation between the two clinical cohorts. In another study, GRS was further validated to show significant discriminatory power for identifying LV outflow tract obstruction in patients with HCM (19). Based on the study from Niu et al., GRS improved obviously after treatment, which suggested that GRS might be an index for evaluating treatment (20). We also provided evidence that the variation and distribution of circumferential strain were similar to those of the radial orientation. The alteration in circumferential direction in this study was consistent with previous investigation (21). This may indicate that motion abilities in radial and circumferential directions of the two diseases were different during the cardiac cycle. Studies have shown that left ventricular myocardium is stratified; fibers in the mid-wall were arranged circumferentially, while such fibers near the apex were sparse (22). This may be the main reason for the significant differences of radial and circumferential strains in basal and midregions. GCS was further expected to be a potential predictor in identifying adverse prognosis, such as malignant arrhythmia (23). LV global systolic functional parameter was correlated the best to circumferential shortening in our data, followed with radial thickening. Extent of hypertrophy (LVMi) revealed an independent role in impacting GCS and GRS in both the HCM and HHD subjects. Another hypertrophy indicator (IVST max) was also proven to be associated with circumferential and radial strains merely in HCM subjects. Previous studies have also shown that myocardial strain values are significantly impaired in hypertensive patients, which are consistent with this study and may offer a preventative strategy before LVEF abnormalities (18).

Longitudinal strain refers to systolic shortening of LV wall relative to its length and it was proven to be a surrogate of subendocardial fibrotic changes in the late stage of HHD disease (24). Based on a previous study, more impaired GLS was measured in patients with HHD compared with patients with HCM (8, 25). But, we merely found a significant difference of longitudinal strain between the two groups in the LV basal segment; no obvious differences were detected in the mid and apical segments. This may be explained by us considering the basal, mid, and apical segments together, which may have diluted the regional variation. Another study also explained the discrepancy above. They reported that longitudinal strain attenuation was unable to provide significant distinguishable accuracy for patients with HCM and HHD with maximal LVWT ≥ 15 mm (7), which was the same condition in this study. Thus, impaired myocardial deformation in the longitudinal direction indicated impaired muscle fibers within the subendocardial region (24).

Left ventricular torsion provides the difference in rotation angle between the base and apex (13). Investigations examining the diagnostic potential of LV torsion were scarce in differentiating between HCM and HHD previously (26). According to this study, LV torsion was higher in the HCM group than that in the HHD group and, therefore, the assessment of LV torsion was shown to provide discriminative ability between patients with HCM and HHD. Increased LVEF was also found in patients with HCM, which was consistent with a previous study (27). The deformation parameters (GLS, GRS, GCS, and torsion) showed underlying effects on contractile function for both the cardiac hypertrophied entities. LVEF alone has previously been insufficient to represent normal deformation ability (28), as most forms of LVH show preserved LVEF until late stages of the disease progression (29). Decreased strain and LV torsion may be able to characterize cardiac pump function in the early phase of disease progression.

Cardiovascular magnetic resonance has the advantage of tissue characterization, which provides valuable diagnostic information. LGE identifies areas with myocyte necrosis or myocardial fibrosis in clinical practice, which has been shown to be closely associated with segmental dysfunction (30–32). Previous publications have demonstrated more prevalent and extensive LGE in patients with HCM than patients with HHD (33); likewise, our data resonated similarly with this. Indeed, the degree of interstitial fibrosis and scar formation in HCM was higher (5). Further comparison regarding LGE quantification (% LGE) in mIVS facilitated its significant differential value, showing that mid-wall fibrosis was a significant discriminator (7). The presence of LGE has also been included in risk prediction modeling for sudden cardiac death in patients with HCM (31). Furthermore, the combination of myocardial strain and LGE provided the highest accuracy rate for differentiation of HHD and HCM. Thus, CMR-derived LGE and myocardial strain measurements offer novel diagnostic significance in differentiating HCM and HHD. As treatment and prognosis of the two hypertrophied cardiac diseases are totally different, CMR has the potential to improve the diagnostic accuracy and facilitate treatment guidance in HCM and HHD.

## Limitations

There were several limitations in this study. First, as only a small cohort of patients was included in this study, larger studies are required to validate our findings. Second, subgroup evaluation may be a promising direction to analyze the differences among diverse phenotypes of HCM and HHD, such as the classification of patients with HCM according to LV outflow tract gradients. Third, we only analyzed systolic strain parameters, which already offered diagnostic value. Diastolic dysfunction was not considered in this study and it might offer new insights to characterize the two diseases. Fourth, a prospective study with the inclusion of genetic findings may help us to better understand the interplay between the genotype and phenotype in these cohorts. Last, the predictive value of myocardial strain for adverse cardiovascular events was not studied due to lack of follow-up.

## Conclusion

Cardiovascular magnetic resonance feature tracking-derived myocardial strain parameters could provide valuable methods for differentiating HCM from HHD. LV strain and torsion were closely related to LVEF, IVST max, and LVMi. LGE (mIVS) has shown the ability of discriminating between HCM and HHD. The combination of LV GRS and LGE (mIVS) achieved the highest diagnostic value. CMR strain analysis may contribute to improve the diagnostic accuracy and facilitate treatment guidance in hypertrophied cardiac diseases.

## Data Availability Statement

The raw data supporting the conclusions of this article will be made available by the authors, without undue reservation.

## Ethics Statement

The studies involving human participants were reviewed and approved by KY2021-132. The patients/participants provided their written informed consent to participate in this study.

## Author Contributions

SL and GW designed the study and wrote the manuscript. XW, YeL, and YZ postprocessed the images. XG, JC, and BX collected the data. ZW and YuL analyzed the data. JT and BY supervised the study. All the authors have read and approved the final version of the manuscript.

## Funding

This study was supported by the National Natural Science Foundation of China (Grant No. 82100529/XG) and the Fund of Key Laboratory of Myocardial Ischemia, Ministry of Education (KF201910/YL).

## Conflict of Interest

The authors declare that the research was conducted in the absence of any commercial or financial relationships that could be construed as a potential conflict of interest.

## Publisher's Note

All claims expressed in this article are solely those of the authors and do not necessarily represent those of their affiliated organizations, or those of the publisher, the editors and the reviewers. Any product that may be evaluated in this article, or claim that may be made by its manufacturer, is not guaranteed or endorsed by the publisher.
